# Insights Into the Effect of Rice Stripe Virus P2 on Rice Defense by Comparative Proteomic Analysis

**DOI:** 10.3389/fmicb.2022.897589

**Published:** 2022-06-07

**Authors:** Zihang Yang, Hehong Zhang, Xiaoxiang Tan, Zhongyan Wei, Caiyi Wen, Zongtao Sun, Bingjian Sun, Jianping Chen

**Affiliations:** ^1^College of Plant Protection, Henan Agricultural University, Zhengzhou, China; ^2^State Key Laboratory for Managing Biotic and Chemical Threats to the Quality and Safety of Agro-products, Key Laboratory of Biotechnology in Plant Protection of Ministry of Agriculture and Zhejiang Province, Institute of Plant Virology, Ningbo University, Ningbo, China

**Keywords:** rice, rice stripe virus, RSV P2, differentially expressed proteins, proteome

## Abstract

Rice stripe virus (RSV) has a serious effect on rice production. Our previous research had shown that RSV P2 plays important roles in RSV infection, so in order to further understand the effect of P2 on rice, we used Tandem Mass Tag (TMT) quantitative proteomics experimental system to analyze the changes of protein in transgenic rice expressing P2 for the first time. The results of proteomics showed that a total of 4,767 proteins were identified, including 198 up-regulated proteins and 120 down-regulated proteins. Functional classification results showed that differentially expressed proteins (DEPs) were mainly localized in chloroplasts and mainly involved in the metabolic pathways. Functional enrichment results showed that DEPs are mainly involved in RNA processing and splicing. We also verified the expression of several DEPs at the mRNA level and the interaction of a transcription factor (B7EPB8) with RSV P2. This research is the first time to use proteomics technology to explore the mechanism of RSV infection in rice with the RSV P2 as breakthrough point. Our findings provide valuable information for the study of RSV P2 and RSV infection mechanism.

## Introduction

Rice stripe disease is an important disease in rice and has greatly endangered rice production, occurring mainly in the temperate and subtropical areas of East Asia (Wu et al., 2009; Otuka, 2013; Huang et al., 2019). Diseased plants often show chlorosis and necrosis on newly developed leaves, followed by stunted plant development, malformed heading, and little fruiting (Zheng et al., 2013; Sun et al., 2016). The disease is caused by rice stripe virus (RSV), which is transmitted by the small brown planthopper (*Laodelphax striatellus*) (Wang et al., 2008). Under natural conditions, RSV only infects graminaceous plants, but under the laboratory conditions *Nicotiana benthamiana* can be infected by mechanical inoculation (Goodin et al., 2008; Arif et al., 2020). RSV is a member of the genus *Tenuivirus* (Phenuiviridae) and its virus particles are non-enveloped filaments with diameters ranging from 3 to 8 nm (Morales and Jennings, 2011; Xu et al., 2012).

The genome of RSV consists of four single-stranded RNAs (ss RNA) (Falk and Tsai, 1998). The largest RNA, RNA1, encodes a single protein on the complementary strand while the other three RNAs each have an ambisense coding strategy, with one open reading frame (ORF) in the virion sense at the 5′ end of the RNA (encoding proteins P2–P4) and one ORF on the complementary strand (encoding proteins Pc2–Pc4) (Wei et al., 2009; Lian et al., 2011; Cho et al., 2013; Zhao et al., 2018). The nucleotide sequences of all genome segments of RSV have been determined, and some biological functions of the seven proteins encoded have been identified, even if rather poorly understood. The single large ORF of RNA1 encodes a 337-kDa RNA-dependent RNA polymerase (Wu et al., 2020). Pc2 (94 kDa) participates in vector transmission by overcoming insect barriers (Yao et al., 2014; Lu et al., 2019). It occurs in the salivary glands and midgut epithelial cells of planthoppers and can co-accumulate with P4 protein in the midgut of poisonous insects (Liang et al., 2005; Zhao et al., 2012). P3 (23.9 kDa) is a silencing suppressor that competes with host protein for double-stranded and single-stranded siRNA to prevent the formation of silencing complex (Xiong et al., 2009; Shen et al., 2010). Pc3 (35 kDa) is a nucleocapsid protein and believed to play a key role in the transmission of RSV through insect ovary (Huo et al., 2014; Liu et al., 2018). P4 (20.5 kDa) is a non-structural disease-specific protein (Kong et al., 2014; Zheng et al., 2017). In infected plants, Pc4 (32 kDa) is mainly distributed near or within the cell wall and can support the cell-to-cell transport of movement-defective potato virus X, and is therefore believed to act as a movement protein (Xiong et al., 2008; Hiraguri et al., 2011). The object of this study, RSV P2, has weak silencing suppressor activity and promotes systemic movement of the virus through its interaction with rice gene silencing suppressor 3 and fibrillarin (Du et al., 2011; Zheng et al., 2015, 2020). The most recent research findings in our laboratory show that RSV P2 interacts with auxin response factor 17 (OsARF17) and impedesDNA binding ability of OsARF17 in rice (Zhang et al., 2020). Besides that, RSV P2 can also target key modules of the jasmonic acid (JA) pathway to inhibit the JA pathway (Li et al., 2021).

Tandem Mass Tag (TMT) technology is a common differential proteomics technology, which has been widely used in animal and plant biology and response to disease and stress (Soares et al., 2009; Yue et al., 2018; Liu X. et al., 2019; Noberini and Bonaldi, 2019; Zhu et al., 2019). Although there have been several proteomics studies of RSV in rice (Wang et al., 2015; Zhao et al., 2019), the effect of RSV P2 on the entire proteome of rice has not been systematically investigated. This study aimed to investigate how RSV P2 affects the host plant, by conducting mass spectrometry proteomics analysis of transgenic rice expressing RSV P2.

## Materials and Methods

### Plant Material

Rice cv. *Nipponbare* (NIP) was used as a background for the transgenic plants expressing the RSV P2 fused to a GFP tag (OEP2) (Biorun, Wuhan, China).

### Experimental Design

**FIGURE 1:**
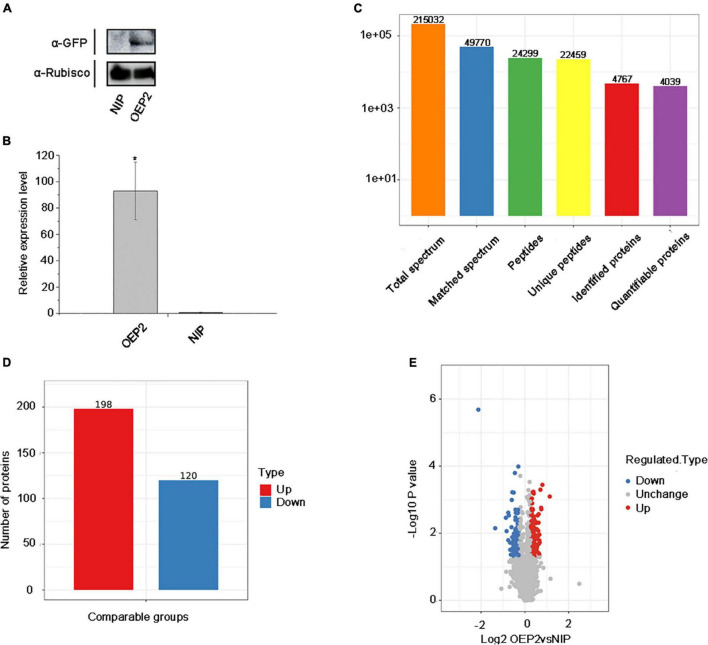
Overview of protein identification and sample repeatability test. **(A)** Detection of RSV P2 by western blot. Rubisco was used as the internal reference gene. **(B)** The relative expression levels of RSV P2 by qRT-PCR. *At the top of columns indicates significant difference at *P* ≤ 0.05 (*n* ≥ 3) by Fisher’s least significant difference tests. *OsUBQ5* was used as the internal reference gene. **(C)** Basic statistics of the mass spectrum data results. **(D)** Histogram of the number distribution of DEPs in different comparison groups. **(E)** Quantitative volcano diagram of DEPs.

Thirty seeds of NIP and OEP2 were randomly selected and cultivated to 2-3 leaf stage. Next, we divided 30 plants into three groups with 10 in each group as one replicate and cut 5 g new leaves, which are placed in 50 ml RNA-free EP tube for protein extraction. Before extracting protein from samples, we have tested the samples by Western blot and qRT-PCR to ensure the expression of RSV P2-GFP in transgenic rice (Figures 1A,B).

### Protein Extraction

The sample was ground to a powder in liquid nitrogen and then transferred to a 5 mL centrifuge tube. Four volumes of lysis buffer [8 M urea (Sigma), 1% Triton-100, 10 mM dithiothreitol (Sigma), and 1% Protease Inhibitor Cocktail (Calbiochem)] was added to the powder, followed by sonication three times on ice using a high intensity ultrasonic processor (Scientz). The remaining debris was removed by centrifugation at 20,000 *g* at 4°C for 10 min. Finally, the protein was precipitated with cold 20% TCA for 2 h at -20°C. After centrifugation at 12,000 *g* 4°C for 10 min, the supernatant was discarded. The remaining precipitate was washed three times with cold acetone. The protein was redissolved in 8 M urea and the protein concentration was determined with a BCA kit (Beyotime, Shanghai, China) according to the manufacturer’s instructions.

### Western Blot

Total protein samples from rice leaves were extracted with 9 sodium dodecyl sulfate (SDS) lysis buffer (100 mM Tris-HCL (pH 6.8), 20% SDS and 2% β-Mercaptoethanol), analyzed on 12% SDS-polyacrylamide gel electrophoresis (SDS-PAGE) gels, and then transferred to the PVDF membrane which had been activated with methanol for 15s before use (Xie et al., 2018). Anti-GFP (Transgen) monoclonal antibody was used to detect the expression of RSV P2 in transgenic rice.

### Measurements of PSII Photochemical Efficiency

Fifteen seeds of NIP and OEP2 were randomly selected and cultivated to 2–3 leaf stage. Then, the rice seedlings were placed in the dark for at least 30 min, and the new leaves of all rice seedlings were cut for measurements of PSII photochemical efficiency. Imaging-PAM (IMAG-MAXI, Germany) was used for measurements of PSII photochemical efficiency under the condition of photosynthetically active radiation (PAR) 141 (Bi et al., 2019).

### RNA Isolation and qRT-PCR

Trizol reagent (GenScript) was used to extract total RNA. The extracted RNA was reverse transcribed by HiScript^®^II Q RT SuperMix for qPCR (Vazyme). qRT-PCR was performed on a Fluorescence quantitative PCR instrument with Hiff^@^ qPCR SYBR^@^ Green Master Mix (YESSEN). The *OsUBQ5* gene (AK061988) was used for the internal control and normalization to calculate fold changes in gene expression (He et al., 2017). At least three biological replicated samples were used.

### Trypsin Digestion

For digestion, the protein solution was reduced with 5 mM dithiothreitol for 30 min at 56°C and alkylated with 11 mM iodoacetamide (Sigma) for 15 min at room temperature in darkness. The protein sample was then diluted by adding 100 mM TEAB (Sigma) to urea concentration less than 2 M. Finally, trypsin (Promega) was added at 1:50 trypsin-to-protein mass ratio for the first digestion overnight and 1:100 trypsin-to-protein mass ratio for a second 4 h -digestion.

### TMT/iTRAQ Labeling and HPLC Fractionation

After trypsin digestion, peptides were desalted by Strata X C18 SPE column (Phenomenex) and vacuum-dried. Peptides were reconstituted in 0.5 M TEAB and processed according to the manufacturer’s protocol for the TMT kit/iTRAQ kit (Thermo Fisher Scientific). Briefly, one unit of TMT/iTRAQ reagent was thawed and reconstituted in acetonitrile. The peptide mixtures were then incubated for 2 h at room temperature and pooled, desalted and dried by vacuum centrifugation.

The tryptic peptides were fractionated into fractions by high pH reverse-phase HPLC using Thermo Betasil C18 column (5 μm particles, 10 mm ID, 250 mm length). Briefly, peptides were first separated with a gradient of 8–32% acetonitrile (pH 9.0) over 60 min into 60 fractions. Then, the peptides were combined into 6 fractions and dried by vacuum centrifugation.

### LC-MS/MS Analysis

The tryptic peptides were dissolved in liquid chromatography solvent A and separated using the EASY-nLC 1000 UPLC system. Solvent A is an aqueous solution containing 0.1% formic acid (Fluka) and 2% acetonitrile (Fisher Chemical). Solvent B is an aqueous solution containing 0.1% formic acid and 90% acetonitrile. Liquid phase gradient settings were: 0–23 min, 9–26% B; 23–32 min, 26–38% B; 32–36 min, 38–80% B; 36–40 min, 80% B, flow rate maintained at 500 NL/min.

The peptides were subjected to NSI source followed by tandem mass spectrometry (MS/MS) in Q ExactiveTM Plus (Thermo) coupled online to the UPLC. The electrospray voltage applied was 2.0 kV. The m/z scan range was 350–1,800 for full scan, and intact peptides were detected in the Orbitrap at a resolution of 70,000. Peptides were then selected for MS/MS using NCE setting of 28 and the fragments were detected in the Orbitrap at a resolution of 17,500. A data-dependent procedure alternated between one MS scan followed by 20 MS/MS scans with 15.0 s dynamic exclusion. Automatic gain control (AGC) was set at 5E4. Fixed first mass was set as 100 m/z.

In this experiment, 215,032 secondary spectra were obtained by mass spectrometry. After searching the protein theoretical data database, the number of available secondary spectrum was 49,770, and the utilization rate was 23.1%. A total of 24,299 peptides were identified by spectrogram analysis, of which the specific peptide was 22,459. A total of 4,767 proteins were identified, of which 4,039 were quantifiable (quantifiable proteins indicate that at least one comparison group has quantitative information).

In this work, the quantitative value of each sample in multiple repetitions was obtained through multiple repeated experiments of total protein quantification. The first step is to calculate the differential expression of the protein between the two samples in the comparison group. First, calculate the average of the quantitative value of each sample in multiple replicates, and then calculate the ratio of the average between the two samples, which is taken as the final differential expression of the comparison group. The second step is to calculate the significance *P*-value of the differential expression of the protein in the two samples. First, take the relative quantitative value of each sample to log 2 (to make the data conform to the normal distribution), and then use the two-sample two-tailed T test method to calculate p-value. When *P*-value < 0.05, the change in differential expression exceeding 1.2 is used as the change threshold for a significant up-regulation, and less than 1/1.2 is used as the change threshold for a significant down-regulation.

### Database Search

The resulting MS/MS data were processed using Maxquant search engine (v.1.5.2.8^1^). Tandem mass spectra were searched against the human uniprot database concatenated with reverse decoy database. Trypsin/P was specified as cleavage enzyme allowing up to 4 missing cleavages. The mass tolerance for precursor ions was set as 20 ppm in First search and 5 ppm in Main search, and the mass tolerance for fragment ions was set as 0.02 Da. Carbamidomethyl on Cys was specified as fixed modification and acetylation modification and oxidation on Met were specified as variable modifications. FDR was adjusted to <1% and minimum score for modified peptides was set >40.

### Protein Classification and Bioinformatics Analysis

GO annotation proteome was derived from the UniProt-GOA database (v.5.14-53.0^2^); The subcellular localization of the protein was categorized according to the annotations of Wolfpsort (v.0.2^3^); KEGG database was used to annotate protein pathways: Firstly, using KEGG online service tools KAAS (v.2.0^4^) to annotated protein’s KEGG database description, then mapping the annotation result on the KEGG pathway database using KEGG online service tools KEGG mapper (V2.5^5^); InterPro (http://www.ebi.ac.uk/interpro/) is a database that integrates diverse information about protein families, domains and functional sites, and makes the information freely available to the public via Web-based interfaces and services; Functional enrichment of proteins used a software Perl module (v.1.31^6^). The database accession or sequence of all DEPs were searched against the STRING database (v.10.1) containing the physical and functional relationships of protein molecules for protein-protein interactions.

### Yeast Two Hybrid System

The coding sequence (cds) of B7EPB8, Q8LH59 and Q0DP16 (Uniprot ID) were cloned into the pGADT7 (activation domain) and the cds of RSV P2 was cloned into the pGBKT7 (binding domain). These structurally corresponding vectors were co-transformed into AH109 yeast strain. The transformed AH109 yeast strain was grown on synthetic defined SD medium lacking Leu and Trp (SD-LT), then spotted on selective SD-media lacking Ade, His, Leu and Trp (SD-A-H-L-T) and grown at 30°C. The primers used are listed in Supplementary Table 1.

The technology roadmap is shown in Supplementary Figure 4.

## Results

### Detection of Transgenic Rice Expressing RSV P2 and Sample Repeatability

To examine the effect of RSV P2 in rice plants *in vivo*, we used transgenic technology to introduce RSV P2 fused with green fluorescent protein (GFP) into *Nipponbare* (NIP) and named it OEP2. So as to ensure the rigor and accuracy of the experiment, we performed three biological repetitions on the samples of NIP and OEP2, and used western blot and qRT-PCR to test the samples for RSV P2 (Figures 1A,B). The primers for qRT-PCR detection of RSV P2 are shown in Supplementary Table 1. Three statistical methods were then used successfully to demonstrate the repeatability of the quantitative protein measurements: principal component analysis, relative standard deviation and Pearson’s correlation coefficient (Supplementary Figures 1A–C).

### Protein Profiling of Transgenic Rice Overexpressing RSV P2

In the proteomic analysis, 215,032 secondary spectra were obtained by TMT/iTRAQ labeling, HPLC fractionation and LC-MS/MS analysis. The number of available spectra was 49,770 and the utilization rate 23.1%. 24,299 peptide segments were identified by spectral analysis, of which 22,459 were specific. A total of 4,767 proteins were identified, of which 4,039 were quantifiable (Figure 1C). Detailed information of identified peptides pertinent to detected proteins is provided in Supplementary Table 2. The lengths of most identified peptides were 7–20 amino acids and most proteins (Supplementary Figures 2A,B).

Quantitative data on the presence of proteins was obtained from the repeat experiments and then analyzed statistically to identify differentially expressed proteins (DEPs). Statistical significance was calculated using the two-sample two-tailed *t*-test and when *p*-value < 0.5, the fold change (FC) of protein was used to represent differential expression of the protein. To understand the functions of these DEPs, the proteins were then classified and annotated using Gene Ontology (GO), subcellular localization, Clusters of Orthologous Groups of proteins (COG), KEGG pathway, predicted functional domains and cluster analysis.

### The Effect of RSV P2 on the Whole Rice Proteome

A total of 198 up-regulated proteins (FC > 1.2) and 120 down-regulated proteins (FC < 0.8) were identified in the transgenic rice expressing RSV P2 compared with the control group (Figures 1D,E). Of these, 14 proteins were upregulated over 1.5-fold and 12 proteins were downregulated over 1.5-fold (Table 1). Detailed information of all the DEPs is provided in Supplementary Table 3.

**TABLE 1:** Characteristics of the significantly differentially expressed proteins.

Protein accession	Gene name	Protein description	Ratio	Type	MW (kDa)
A0A0P0XI80	Os08g0519050	Myosin heavy chain-like protein(predicted)	2.197	Up	22.047
Q6ZD29	Os08g0374000	Bet_v_1 domain-containing protein(predicted)	1.584	Up	16.548
Q8LH59	MYBS1	Transcription factor MYBS1	1.585	Up	31.912
Q84QV5	OJ1191_A10.120	FK506-binding protein 2-1(predicted)	1.595	Up	113.46
Q9SLY8	CRO1	Calreticulin OS = *Oryza sativa* subsp.	1.683	Up	48.308
Q53WK4	NAP1;2 9	Nucleosome assembly protein 1;2	1.525	Up	41.683
Q10NM5	LOC_Os03g15870	50S ribosomal protein L4, chloroplast, putative, expressed	1.583	Up	34.52
Q7XCL2	OSJNBb0015I11.23	Ubiquitin family protein, expressed(predicted)	1.545	Up	59.314
Q10N44	Os03g0283200	Protein IN2-1 homolog A	1.558	Up	27.299
Q6YY42	Os02g0589000	Lecithin-cholesterol acyltransferase-like 1(predicted)	1.635	Up	49.017
Q10MP7	LOC_Os03g18850	Bet_v_1 domain-containing protein(predicted)	1.54	Up	17.173
Q0DJT2	Os05g0230900	Lactoylglutathione lyase (Fragment)	1.742	Up	32.492
Q339E0	LOC_Os10g22100	Expressed protein	1.671	Up	77.187
Q6H759	P0476H10.30-1	Copper chaperone homolog CCH	1.547	Up	13.094
P38419	CM-LOX1	Lipoxygenase 7, chloroplastic	0.601	Down	102.82
Q0JD85	FBN5	Fibrillin protein 5 homolog	0.655	Down	30.617
Q2QNT0	LOC_Os12g36850	Bet_v_1 domain-containing protein(predicted)	0.591	Down	16.981
Q8RZ83	Os01g0667600	Ras-related protein RABA1f(predicted)	0.628	Down	24.444
Q7F2P0	Os01g0382000	Pathogenesis-related protein PR1b(predicted)	0.56	Down	17.462
Q7XR61	MTK1	Methylthioribose kinase 1	0.548	Down	48.413
Q7XXD3	Os04g0175600	Probable inactive methyltransferase Os04g0175900(predicted)	0.39	Down	40.594
Q93WM2	Os01g0374000	Putative glutathione S-transferase GSTF1 isoform X1(predicted)	0.229	Down	24.242
A0A0N7KGD8	Os02g0830100	Oligopeptidase A-like(predicted)	0.59	Down	78.594
Q6K7P8	Os02g0828200	CBFD_NFYB_HMF domain-containing protein(predicted)	0.626	Down	47.244
Q94CU3	P0423B08.43-1	Uricase	0.642	Down	34.503
Q0J360	CIN7	Beta-fructofuranosidase, insoluble isoenzyme 7	0.66	Down	65.489

**FIGURE 2:**
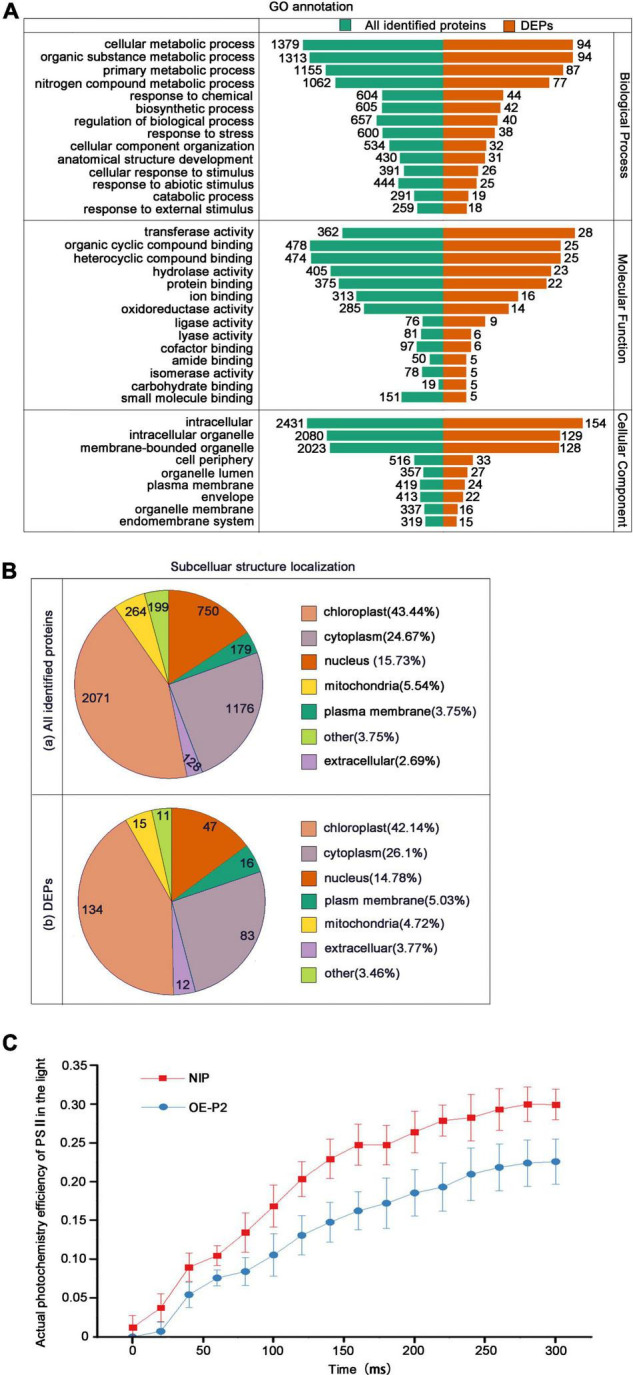
Classification of all identified proteins and DEPs, and measurements of PSII photochemical efficiency. **(A)** GO analysis of all identified proteins and DEPs. All proteins were classified by GO annotation based on their cellular component, molecular function, and biological process. **(B)** Subcellular locations of identified proteins (a) and DEPs (b). **(C)** Measurements of PSII actual photochemical efficiency of NIP and OEP2 in the light. At least 15 NIP or OEP2 were used for photochemical determination.

Through GO annotation, all identified proteins and DEPs can be allocated to three categories representing their biological function: Biological Process, Cellular Component and Molecular Function. More detailed identification is provided by the GO secondary annotation classification. The total numbers of identified proteins and DEPs were similar in biological process and cellular component, with fewer in the molecular function category. In the biological process category, most of the identified proteins and DEPs were in the metabolic process subcategory. Full details are provided in Figure 2A.

Subcellular localization showed that both the identified proteins (Figure 2B(a)) and the DEPs (Figure 2B(b)) were widely localized in chloroplasts (> 40%), cytoplasm (25–26%) and nucleus (15–16%). Due to that the most identified proteins and DEPs were located in chloroplasts, we detected the PSII photochemical efficiency of NIP and OEP2. The results revealed that PSII maximum photochemical efficiency showed no significant difference in these samples (Supplementary Figure 1D), but PSII actual photochemical efficiency of OEP2 is significantly lower than that of NIP (Figure 2C), suggesting that RSV P2 may directly affect the function of chloroplasts.

**FIGURE 3:**
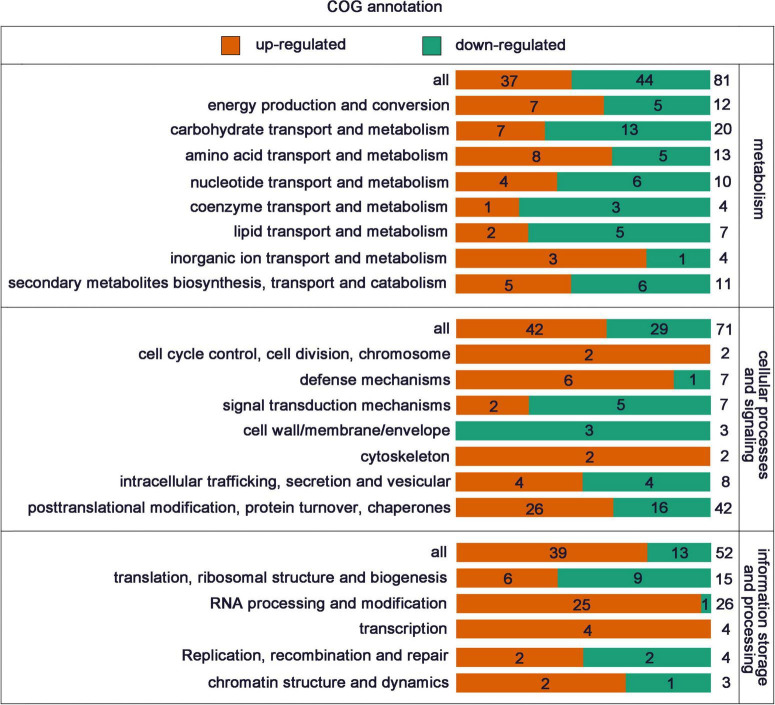
COG functional classes of all the DEPs.

COG analysis allocates the proteins into three groups: metabolism (81 DEPs), cellular processes and signaling (71 DEPs), information storage and processing 52 DEPs (Figure 3). In the cellular processes and signaling, 46 of 71 DEPs were related to “posttranslational modification, protein turnover, chaperones.” In the information storage and processing, 26 of 52 DEPs were related to “RNA processing and modification” and all but one of these was up-regulated. The annotation information of all identified proteins is provided in Supplementary Table 5.

### Functional Enrichment and Cluster Analysis of DEPs

**FIGURE 4:**
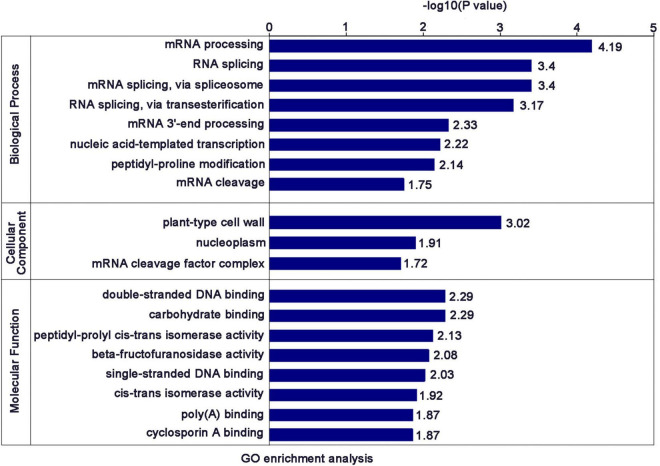
Significantly enriched GO terms of the DEPs.

To investigate whether the DEPs have a significant enrichment trend in certain functional types, the DEPs in each treatment group were analyzed by GO classification, KEGG pathway and protein domain enrichment analysis. Cluster analysis based on GO enrichment is also carried out from three aspects: biological process, cellular component and molecular function. In terms of biological process, the DEPs were mainly enriched in “mRNA processing,” “RNA splicing,” “mRNA splicing, via spliceosome,” “RNA splicing, via transesterification reaction,” “mRNA 3′-end processing,” “nucleic acid-templated transcription,” “peptidyl-proline modification,” and “mRNA cleavage.” In terms of cellular component, the DEPs were mainly enriched in “plant-type cell wall, “nucleoplasm,” “mRNA cleavage factor complex.” In terms of molecular function, the DEPs were mainly enriched in “double-stranded DNA binding,” “carbohydrate binding,” “peptidyl-prolyl cis-trans isomerase activity,” “beta-fructofuranosidase activity,” “single-stranded DNA binding,” “cis-trans isomerase activity,” “poly(A) binding,” “cyclosporin A binding” (Figure 4).

**FIGURE 5:**
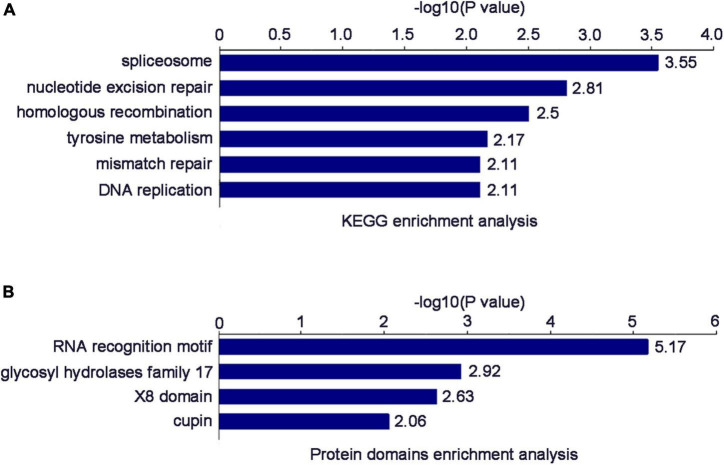
**(A)** Significantly enriched KEGG terms of the DEPs. **(B)** Significantly enriched Protein domain terms of the DEPs.

The enrichment results of the KEGG pathway showed that the DEPs were mainly enriched in “spliceosome,” “nucleotide excision repair,” “homologous recombination,” “tyrosine metabolism,” “mismatch repair,” and “DNA replication” (Figure 5A). The enrichment results of protein domain showed that the DEPs were mainly enriched in “RNA recognition motif,” “glycosyl hydrolases family 17,” “X8 domain,” and “cupin” (Figure 5B).

### qRT-PCR Verification on Some DEPs Identified by TMT Quantitative Proteomics

**FIGURE 6:**
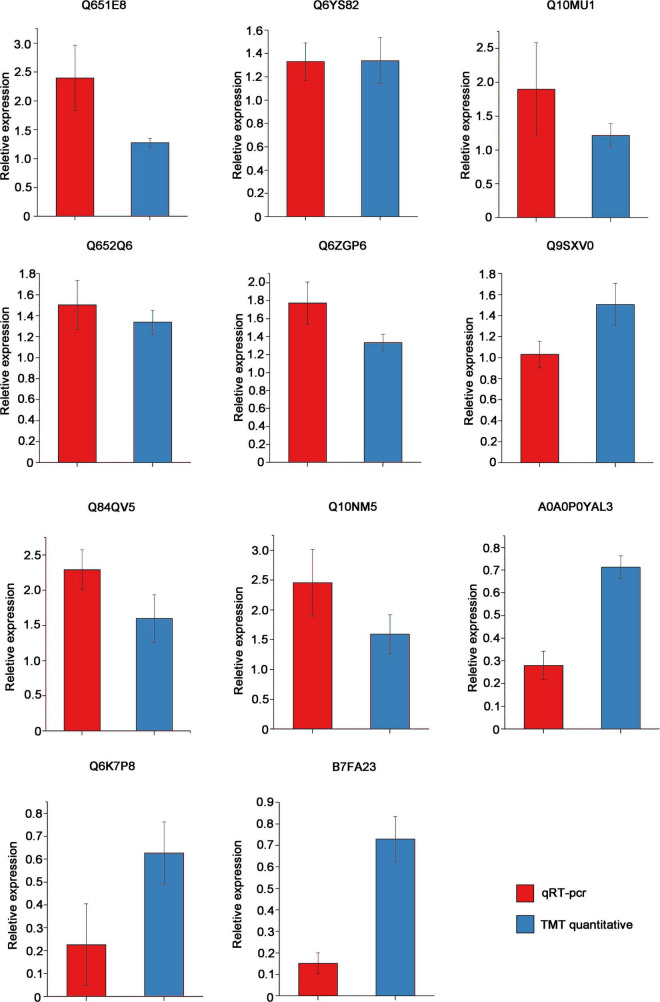
Results of qRT-pcr to veify the mRNA expression of 11 proteins that had been identified as DEPs by TMT quantitative proteomics in OEP2 relative to NIP. *OsUBQ5* was used as the internal reference gene. Each sample has at least 3 biological replicates.

To verify the accuracy of our proteomics results, we randomly selected 11 proteins [Q651E8, Q6YS82, Q10MU1, Q652Q6, Q6ZGP6, A0A0P0YAL3, Q6K7P8, B7FA23, Q9SXV0, and Q10NM5 (Uniprot ID)] for qRT-PCR verification. The primers for qRT-PCR detection and basic information of these 11 proteins are shown in Supplementary Tables 1, 4. The expression of these proteins at the mRNA level was consistent with the results of TMT quantitative proteomics. Compared with NIP, the transcript levels of Q651E8, Q6ZGP6 and Q10NM5 were greatly elevated, while the transcript levels of A0A0P0YAL3, Q6K7P8, and B7FA23 were significantly decreased (Figure 6). Among these proteins, Q84QV5 and Q10NM5 were significantly up-regulated at both mRNA leveland protein level. Q10NM5 is 50S ribosomal protein L4. In animals, the ribosomal protein L4 has been identified as a putative interacting partner of viral VP3 protein, regulating the replication of infectious bursal disease virus (Chen et al., 2016; He et al., 2016). Therefore, it would be interesting to investigate whether the 50S ribosomal protein L4 of rice interacts with RSV P2 to regulate RSV replication.

To demonstrate that these DEPs were specifically caused by RSV P2 protein, we selected Q6K7P8, Q6YS82, and Q10MU1 for qRT-PCR verification in NIP, OEP2, and GFP-Turbo transgenic rice (in which GFP was fused to a Turbo protein). The results showed that compared with NIP, these genes were significantly changed in OEP2 plants, but there was no significant change in GFP-Turbo (Supplementary Figure 3). Hence, these results suggested that these DEPs were indeed caused by P2 protein.

### Testing the Interaction Between Differentially Expressed Transcription Factors and RSV P2 by Y2H

**FIGURE 7:**
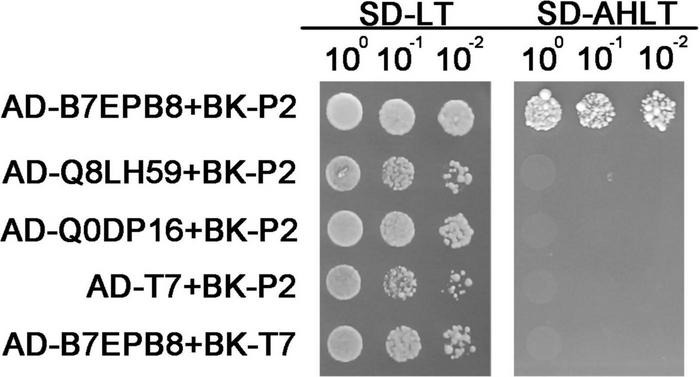
Verification of interaction between B7EPB8, Q8LH59, Q0DP16 and RSV P2 by Y2H. Transformed yeast cells were grown on SD-Leu-Trp and SD-Ade-His-Leu-Trp medium.

Our previous studies had shown that RSV P2 could directly interact with OsARF17, hindering DNA binding ability of OsARF17, and weakening the disease resistance of rice (Zhang et al., 2020; Li et al., 2021). Because that work had shown that transcription factors are very important targets for RSV P2 in overcoming plant defense, we tested the transcription factors in all the DEPs to determine whether they could directly interact with RSV P2. Of all the DEPs detected, seven proteins were identified as transcription factors: five up-regulated proteins (Q8LH59, B7EPB8, Q75IZ7, Q6ZGP6, Q8RUI4) and two down regulated proteins (Q69IM4, Q0DP16). Of these, we successfully cloned B7EPB8, Q8LH59 Q0DP16 and used the Y2H system to test for interactions with RSV P2 (Figure 7). B7EPB8 has been reported as a homolog of *Arabidopsis* VIP1 protein, which can directly target genes in JA signaling and metabolic pathways to positively regulate JA levels (Liu D. et al., 2019). In our Y2H assay, only B7EPB8 interacted with RSV P2 and it seems likely that this interaction would act on rice JA pathway and metabolic pathways to affect the defense response of rice.

## Discussion

Proteomics is a collective term for studying all proteins expressed by a cell or even an organism. Nowadays, high throughput proteomic analysis has developed to explore the effect of viral proteins on host plants at the protein level based on mass spectrometry with greater automation and sensitivity (Liu D. et al., 2019). In this study, rice overexpressing RSV P2 was used as material for the first time to analyze the role of RSV P2 in the process of RSV infection of rice through TMT quantitative proteomics technology. This not only provides new ideas for the flexible application of proteomics technology, but also provides basic information for the in-depth exploration of RSV P2.

Plants accumulate an amazing diversity of phytochemicals through metabolic pathways, and virus invasion causes many changes in plant metabolism (Grotewold, 2005; Fernández-Calvino et al., 2014). In our study, GO classification annotation showed that identified proteins and DEPs were mainly “metabolic process”-related protein. KOG classification annotation identified 162 DEPs involved in “metabolic”-related pathways. Among these DEPs, proteins related to adenosine monophosphate (AMP) appeared at a higher frequency in the “metabolic process” of GO classification annotation and “lipid transport and metabolism” pathways of KOG classification annotation. Cyclic AMP is an important second messenger of cell signaling and plays important roles in different physiological processes, including regulation of gene expression, response to hormonal stimulation and plant defense responses (Montminy, 1997; Lee et al., 2005; Sabetta et al., 2019). We screened out four genes related to AMP: two AMP-binding enzyme family proteins (Q2QX58 and B7FA23), AMP-binding domain-containing protein (Q0JKR1), and AMP-binding protein (Q75LB3). Previous reports had shown that a defense associated AMP binding protein OsBIABP1 is involved in the regulation of defense response through salicylic acid (SA)/JA/ethephon signaling pathways (Zhang et al., 2009). These results showed that the AMP-related protein may resist the invasion of RSV by acting on the P2 of RSV, which still needs further research.

Recent virus research has highlighted the important role of chloroplasts in the process of virus infection. Viral infection can cause changes in chloroplast structure, composition, and expression of chloroplast-related proteins. For example, tobacco chloroplast proteins AtpC and RCA interact with tobacco mosaic virus replication-related protein VRCs, thereby increasing tobacco resistance to viruses (Bhat et al., 2013). Peach latent mosaic viroid utilizes host RNA silencing mechanism to silence the chloroplast-targeted heat shock protein 90, leading to leaf chlorosis and reduced photosynthetic activity of chloroplasts (Bhor et al., 2017). C4 protein of tomato yellow leaf curl virus can be transferred from the plasma membrane to the chloroplast and interact with the thylakoid membrane-associated calcium sensing receptor to inhibit the JA pathway (Medina Puche et al., 2020). In addition, interaction of the chloroplast and its factors with virus components or factors have been shown to play significant roles in the replication, movement and symptom development of viruses. For instance, the significant decrease of alfalfa mosaic virus (AMV) accumulation in infected leaves is due to the interaction between the coat protein of AMV and chloroplast protein PsbP (PSII oxygen-evolving complex protein) (Balasubramaniam et al., 2014); The multifunctional triple gene block protein 1 of the potexvirus Alternanthera mosaic virus has a selective response with the chloroplast β-ATPase of *Arabidopsis thaliana*, which can induce defense responses and reduce viral symptoms (Seo et al., 2014). In this present study, 134 DEPs were predicted to be located in the chloroplast and PSII actual photochemical efficiency of OEP2 is significantly lower than that of NIP, showing that the RSV P2 is closely associated with the chloroplast.

After a preliminary comparison, COG classification annotation showed that 26 DEPs were associated with “RNA processing and modification,” while the biological process of GO enrichment also showed that DEPs were mainly involved in “RNA processing and splicing.” KEGG enrichment suggested that DEPs were mainly involved in the “spliceosome.” These results all strongly point to the involvement of RSV P2 in RNA processing and splicing. As expected, they contain some of the same proteins, including two ribonucloproteins (Q10PA5 and Q5ZDX8), one nuclear cap-binding protein (Q84L14), two RRM domain-containing proteins (Q8LHL0 and A0A0P0 × 3W0), two splicing factors (Q69KL9 and Q8LIB1) and two ATP-dependent RNA helicases (Q7XIR8 and Q84MP1). Surprisingly, homologs of these proteins in other plants have been shown to be involved in gene silencing. For example, In *Arabidopsis*, the combination of nuclear ribonucleoprotein SmD1 with RNAs transcribed from silenced transgenes indicated that SmD1 had a direct role in post-transcriptional gene silencing (Elvira Matelot et al., 2016); The consumption of Ars2, a component of the nuclear RNA cap binding complex, reduced the level of RNA and provided evidence for the role of Ars2 in the regulation of RNA interference (Cao et al., 2019); The absence of the RRM domain in GW182 proteins impairs the silencing activity of GW182 in a miRNA-targeted manner, suggesting that this domain contributes to silencing (Eulalio et al., 2009). The splicing factors ZOP1, STA1, PRP31 and several other splicing-related proteins were both physically and functionally connected, and contributed to transcriptional gene silencing (Du et al., 2015). Many RNA helicases were isolated from the screening of RNA interference factors and regulate gene silencing at almost every level of the RNA interference pathway (Ambrus and Frolov, 2009). Previous studies have shown that the RSV P2 is a silencing suppressor, so we speculate that P2 may promote the infection of RSV by inhibiting gene silencing of host plants, but the specific mechanism needs more in-depth research.

Virus-encoded proteins can control the movement of plant viruses from cell to cell, and plant-type cell wall localization of viral movement proteins is necessary for cell-to-cell movement (Tremblay et al., 2005). In our study, 8 plant-type cell wall-associated proteins were enriched by Cellular Component of GO enrichment, whether P2 participates in the movement of RSV with the help of these eight plant-type cell wall-related proteins still needs further verification. a result that may provide additional evidence that P2 is involved in RSV movement (Zheng et al., 2015).

Molecular Function of GO enrichment analysis revealed that RSV P2 can affect the expression level of 9 carbohydrate binding-related proteins and previous studies have shown that such proteins have antiviral effects. For instance, the θ-defensin retrocyclin 2, a carbohydrate-binding molecule, can inhibit viral fusion and entry through cross-linked membrane glycoproteins (Leikina et al., 2005). Whether these proteins can use this strategy to resist virus invasion by interacting with the P2 needs more in-depth research. Transcription factors are important factors that maintain the expression of genomic functional protein genes and play a significant role under biological stresses, such as viruses (Sharoni et al., 2010; Lee et al., 2012; Nuruzzaman et al., 2013). In our study, we found 7 transcription factors among the DEPs and cloned B7EPB8, Q8LH59 and Q0DP16. Although there are few reports about Q0DP16, but MYB transcription factors are closely involved in abiotic and biotic stress responses in rice and *Arabidopsis* (Dai et al., 2007; Ma et al., 2009; Dubos et al., 2010; Yang et al., 2012). Q8LH59 can respond to some nutritional deficiency signals, is related to the gibberellin acid pathway, and can activate the expression of target genes (Lu et al., 2002; Hong et al., 2012). B7EPB8 is homologous to the *Arabidopsis* VIP1 gene and can interact with PR proteins in yeast. Moreover, the levels of JA and SA are up-regulated in transgenic rice overexpressing B7EPB8. Methyl jasmonic acid (MeJA) can largely induce the expression of B7EPB8 (Liu D. et al., 2019). Our results showed that only B7EPB8 interacted with RSV P2 in a Y2H assay, which indicates that RSV P2 may directly interact with B7EPB8 to interfere with the interaction between PR proteins and B7EPB8, act on the rice JA pathway, weaken rice disease resistance, and enhance the pathogenicity of RSV, which still needs further verification and research.

## Conclusion

In our study, we used TMT quantitative proteomics to conduct comparative proteomic analysis of transgenic rice overexpressing RSV P2. A total of 318 DEPs were identified, including 198 up-regulated proteins and 120 down-regulated proteins. The functional classification showed that these DEPs are mainly located in the chloroplast and mainly participate in metabolic pathways. Functional enrichment analysis showed that DEPs are mainly involved in RNA processing and splicing. We also verified the expression of several DEPs at the mRNA level by qRT-PCR and the interaction of a transcription factor B7EPB8 with RSV P2 by Y2H assay. These results provided fundamental resources for subsequent in-depth research on RSV P2.

## Data Availability Statement

The original contributions presented in the study are included in the article/Supplementary Material, further inquiries can be directed to the corresponding author/s.

## Author Contributions

ZY, CW, BS, JC, and ZS conceived the project and designed the experiments. ZY, HZ, and XT carried out the experiments with assistance from ZW. ZY, BS, JC, and ZS analyzed and discussed the results. ZS wrote the manuscript. All authors contributed to the article and approved the submitted version.

## Conflict of Interest

The authors declare that the research was conducted in the absence of any commercial or financial relationships that could be construed as a potential conflict of interest.

## Publisher’s Note

All claims expressed in this article are solely those of the authors and do not necessarily represent those of their affiliated organizations, or those of the publisher, the editors and the reviewers. Any product that may be evaluated in this article, or claim that may be made by its manufacturer, is not guaranteed or endorsed by the publisher.

## Footnotes

1
http://www.maxquant.org/

2
http://www.ebi.ac.uk/GOA/

3
http://www.genscript.com/psort/wolf_psort.html

4
http://www.genome.jp/kaas-bin/kaas_main

5
http://www.kegg.jp/kegg/mapper.html

6
https://metacpan.org/pod/Text:NSP:Measures:2D:Fisher

## Supplementary Material

Supplementary Figure 1 Sample repeatability test and measurements of PSII photochemical efficiency. **(A)** Two-dimensional scatter plot of quantitative principal component analysis of protein between repeated samples. The better the degree of aggregation between repeated samples, the better the quantitative repeatability. **(B)** Box plot of quantitative RSD distribution of protein between repeated samples. The smaller the overall RSD value, the better the quantitative repeatability. **(C)** Pearson correlation coefficient heat map of protein quantification between two groups. **(D)** Measurements of PSII maximum photochemical efficiency of NIP and OEP2 in the dark. At least 15 NIP or OEP2 were used for photochemical determination.

Supplementary Figure 2 **(A)** The length distribution of peptides was identified by mass spectrometry. **(B)** All identified proteins mass distribution.

Supplementary Figure 3 qRT-PCR detects the gene expression in NIP, OEP2 and GFP-Turbo plants.

Supplementary Figure 4 Technology roadmap.
