# Supplementary figures and images for: Insights Into the Effect of Rice Stripe Virus P2 on Rice Defense by Comparative Proteomic Analysis
Source: Front Microbiol. 2022 Jun 7;13:897589. doi: 10.3389/fmicb.2022.897589 (PMC9209781; doi:10.3389/fmicb.2022.897589)

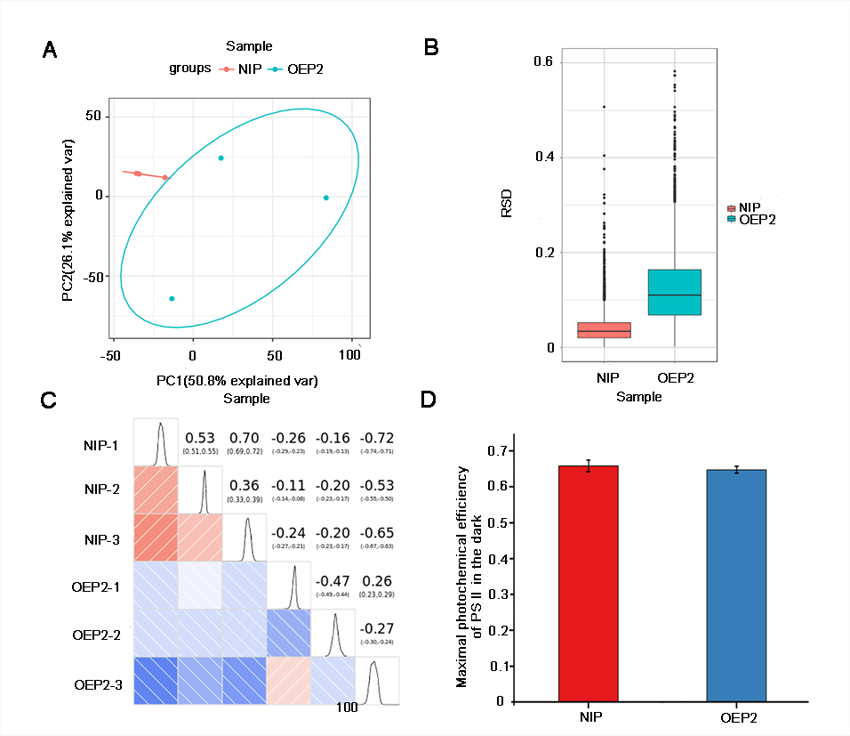

Supplement: Supplementary Figure 1 — Sample repeatability test and measurements of PSII photochemical efficiency. (A) Two-dimensional scatter plot of quantitative principal component analysis of protein between repeated samples. The better the degree of aggregation between repeated samples, the better the quantitative repeatability. (B) Box plot of quantitative RSD distribution of protein between repeated samples. The smaller the overall RSD value, the better the quantitative repeatability. (C) Pearson correlation coefficient heat map of protein quantification between two groups. (D) Measurements of PSII maximum photochemical efficiency of NIP and OEP2 in the dark. At least 15 NIP or OEP2 were used for photochemical determination. [file Image_1.TIFF]

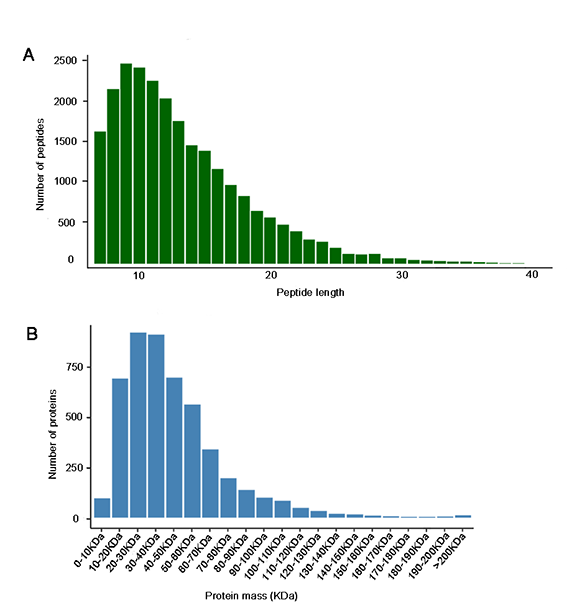

Supplement: Supplementary Figure 2 — (A) The length distribution of peptides was identified by mass spectrometry. (B) All identified proteins mass distribution. [file Image_2.TIFF]

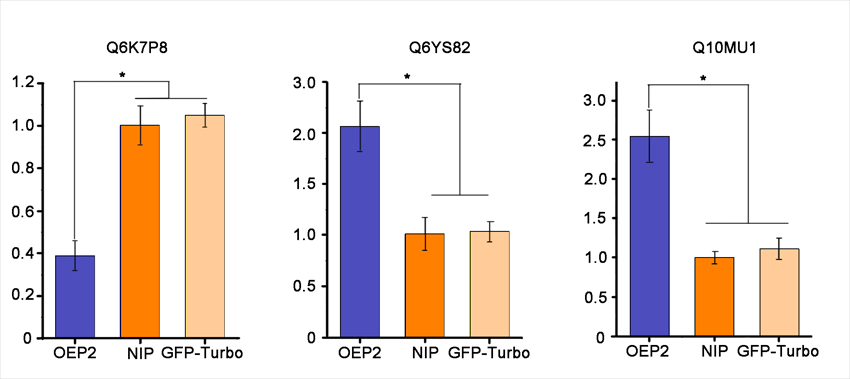

Supplement: Supplementary Figure 3 — qRT-PCR detects the gene expression in NIP, OEP2 and GFP-Turbo plants. [file Image_3.TIFF]

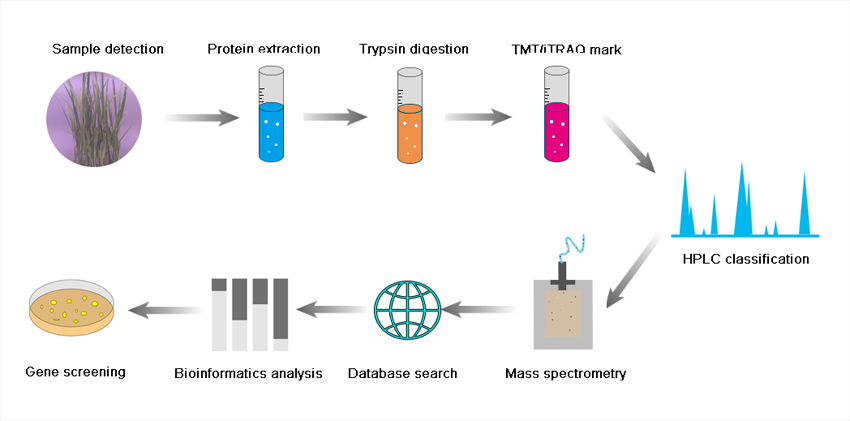

Supplement: Supplementary Figure 4 — Technology roadmap. [file Image_4.TIFF]
